# The benefits of a peer-support group for paediatric rheumatology nurses working in isolation

**DOI:** 10.1093/rap/rkac043

**Published:** 2022-05-19

**Authors:** Heather Rostron, Polly Livermore

**Affiliations:** 1 Children’s Clinical Research Team, Leeds Teaching Hospitals NHS Trust, Leeds; 2 Rheumatology Department, Children’s Hospital NHS Foundation Trust; 3 BRC Great Ormond Street Children’s Hospital NHS Foundation Trust, London, UK

Clinical Nurse Specialists (CNSs) can often feel isolated owing to the intense workload and pressure to keep services going. Detached from ward-based nursing teams and working with huge patient caseloads, CNSs have many competing demands on their time. Rheumatology is no exception, with the role of the Rheumatology CNS being highly complex, involving such skills as joint examination, clinical assessment, IA joint injections, nurse prescribing and, above all, constant patient education to keep those with chronic, disabling health conditions out of hospital. The aim of this editorial is to demonstrate and showcase how the network for paediatric and adolescent rheumatology nurses around the UK has evolved to provide essential, instantaneous support and guidance, particularly for CNSs working in isolation.

Paediatric rheumatology diseases are autoinflammatory conditions diagnosed before a patient’s 16th birthday, which include such conditions as JIA, JDM and JSLE. JIA is the most common of these, with an age-standardized prevalence rate of 43.5 per 100 000 in 2018 in the UK [1]. These conditions have huge impact not only on the child or young person’s quality of life, but also on their family around them.

## Need for a network

Owing to the chronic nature, wide variety and rarity of some of these the conditions seen within children’s rheumatology services, nursing clinical practices can be variable. These factors have the potential to make nurses feel isolated and unconfident in their practice, particularly in secondary health-care centres with perhaps few nurses working in this specialism. Following an advert in the Royal College of Nursing (RCN) Rheumatology Bulletin two decades ago, a national peer support network was set up for nurses working in children’s rheumatology services. Thus, the RCN Children and Young People’s (CYP) Rheumatology Nurses group first met face to face in 2002 in Stratford-Upon-Avon. There were 20 attendees at the inaugural meeting, but this number included some adult rheumatology nurses and allied health professionals who also asked to attend.

Since that first meeting in 2002 and up to March 2020, children’s rheumatology nurses from across the UK have come together annually to network and support each other. Venues have ranged from Edinburgh to Southampton and anywhere in between, often aligning with the British Society for Rheumatology’s (BSR) annual conferences. All meetings were generously funded by pharmaceutical companies, enabling nurses to arrive at lunchtime, network all afternoon, have an evening meal together (sometimes with added fun events, such as a cocktail-making class) and stay overnight, before continuing on until the following lunchtime. The advantage of this format was that individuals had the morning and the following afternoon to travel, hence wherever the meeting was held, it had travel time built in, and this was the only financial outlay for each attendee.

To our knowledge, this UK group of children’s rheumatology nurses is unique in this clinical speciality throughout Europe. Particular strengths of the group include inclusivity for all, regular updates, discussion of local projects and peer support. The group has also allowed for the recruitment of nurses to national multidisciplinary projects, such as representation on conference planning committees and research projects. Another advantage of the group is putting nurses in contact with each other; this is not only invaluable when patients move area, for example attending university in a different city, but also for nurses to be able to contact specific colleagues quickly and easily through shared email addresses. Our group currently straddles both the RCN and BSR, with membership of either not being a compulsory stipulation for membership of this support group.

## Current group

Membership has continued to grow since the early years following the RCN bulletin advert and establishment of the group. Currently, there are >80 paediatric CNSs from 33 centres signed up to receive the group’s emails, with membership changing on a daily basis as nurses join and leave teams. In these 33 centres of which we are aware, there are 14 with only one CNS in paediatric rheumatology, and it is these nurses who have particularly vocalized the support they have received from this group. One recently commented: ‘I don’t want to ask questions that are nursing related or I feel I should know the answer to, with my multi-disciplinary team, but being able to gain a quick non-judgmental response from my nursing colleagues has been a life saver many times’.

Despite being unable to meet in person, the group has adapted and become more dynamic than ever during the coronavirus disease 2019 (COVID-19) pandemic. Changes include meeting online, increased frequency of meetings, invited speakers and support received from BSR. The BSR has hosted virtual meetings, managed the associated administration and prepared agendas. Membership has grown, with 15 new individuals added throughout 2021, perhaps demonstrating an increase in specialist nursing staff in addition to engagement. The formation of a smartphone messaging group was necessary owing to the needs expressed by group members to obtain instant replies; this currently hosts 49 participants.

During the pandemic, the group also welcomed Clinical Research Nurses either delivering children’s rheumatology studies or who had a specialist interest in this area. There are numerous National Institute for Health Research studies running in children’s rheumatology, and the group felt that including Clinical Research Nurses could only lead to greater synergy in striving to improve the care of their patients and families. Furthermore, we also have had a nurse with an interest in rheumatology ask to join the group, in addition to two adult nurses who have an interest in the transition to adult services.

Since March 2020, we have held five virtual meetings, with attendance ranging from 12 to 23. Some meetings were duplicates because different times of the day were offered to nurses according to what worked best for them (e.g. some nurses preferred meeting at 19:00 h as opposed to during the day because they were home schooling or busy clinically). Meetings are set up bi-monthly for 2022.

We examined the emails received during 2021, and they fell into three main themes: sharing of information (such as upcoming BSR meetings/webinars); guidelines (asking for guidelines for certain therapies or treatment pathways); and COVID-19 related (asking for information on vaccines, change in restrictions, and ways of working). All queries were emailed to the whole group, enabling anyone to respond.

## Benefits of virtual support

Most children’s rheumatology CNSs have a telephone advice line or an answerphone with times for patients’ parents to call back with queries. Although this is useful for parents, it can often mean that other health-care professionals struggle to get through quickly on the telephone. An email or Whatsapp message, however, allows CNSs to be able quickly to obtain an answer or receive a guideline for a medication they might not have used before. Especially during the pandemic, when practices were changing very rapidly (e.g. biologic therapies changed from i.v. to s.c. administration), the email and messaging group allowed nurses quickly and safely to ask for support from a peer group who understand the nature of the pressures exactly.

Also, during the pandemic it quickly became apparent that meeting online did, in fact, offer benefits to members. Meeting virtually was easier than arranging time off work; national travel and subsistence costs were no longer required. Nurses were able to drop in during their lunchbreak or to join in during the evenings, when they could concentrate without worrying about the work that needed to be done. In a recent survey of the group, all 50 respondents (100%) said that their roles had changed since the pandemic began, with 36 (72%) saying they had been allowed to work from home, and all 50 also said that patient care had changed [2]. This rapidly changing current way of working, with further isolation from home working, can create huge emotional strains on an ever-stretched workforce [3].

Although this group is not a formal peer support as defined during the pandemic [4], going forwards it is important to capture from members how the group will continue to fulfil its aims and remain fit for purpose for its members [5]. Feedback received so far highlights the benefit that nurses gain from attending these meetings and, although most would like a face-to-face get together as soon as possible, these meetings still have a very clear purpose and successful result.

The administration of such a large dynamic group is a challenge, with new nurses frequently joining and others leaving or retiring; however, the opportunities to learn from the more experienced members is always welcomed. Discussions with the nurses in the Pediatric Rheumatology European Society (PReS) and the EULAR have also begun to identify opportunities to collaborate on shared projects and form a Europe-wide paediatric rheumatology nurses group.

A recent question in the group asked about patient-reported side effects for one of the new biologics after a team switched three patients to it, but had never used it before. Learning from real-life experience of others, rather than trawling through the literature and regurgitating advice on patient information leaflets, allows nurses to be up-to-date and more informed practitioners. In conclusion, this support group is vital to protect the wellbeing of CNSs, especially those who work in isolation from paediatric rheumatology nurse colleagues.
